# Investigating the Causes of Molar Incisor Hypomineralization: A Cross-Sectional Study on Maternal and Child Health Factors

**DOI:** 10.21142/2523-2754-1204-2024-216

**Published:** 2024-11-23

**Authors:** Ozlem Martı Akgün, Ceren Yıldırım, Umit Oflaz, Basak Topaclıoglu

**Affiliations:** 1 Gulhane Faculty of Dentistry, University of Health Sciences. Ankara, Turkey. ozlemmartiakgun@gmail.com, cerenk.yidirim@gmail.com, dtcharon07@gmail.com, basaktopaclioglu@hotmail.com Gulhane Faculty of Dentistry University of Health Sciences Ankara Turkey ozlemmartiakgun@gmail.com cerenk.yidirim@gmail.com dtcharon07@gmail.com basaktopaclioglu@hotmail.com

**Keywords:** molar incisor hypomineralization, enamel, dental health, prenatal care, postnatal nutrition, hipomineralización de molares e incisivos, esmalte, salud dental, cuidado prenatal, nutrición posnatal

## Abstract

**Objectives:**

This study aims to investigate the factors contributing to Molar Incisor Hypomineralization (MIH), with a focus on maternal health during pregnancy, delivery complications, and postnatal health and nutritional factors in children.

**Methods:**

A cross-sectional study was conducted involving detailed surveys completed by mothers regarding their health during pregnancy, delivery experiences, and their children's early health and nutritional history. Clinical examinations of the children were performed to diagnose MIH according to established criteria. Statistical analyses were conducted using IBM SPSS 29.0, including descriptive statistics and correlation analyses to identify associations between MIH and various maternal and child health factors.

**Results:**

The study found that 36.7% of mothers experienced illnesses during pregnancy, with gestational diabetes and hypertension being the most common conditions. Complications during delivery were observed in 3.3% of cases, and 16.7% of mothers had premature births. Common health issues in children included respiratory infections (26.7%), fever (23.3%), and otitis media (16.7%). Notably, dental defects were most prevalent in children with kidney disorders (66.7%), digestive system disorders (45.8%), and severe diarrhea (43.8%). Significant correlations were identified between MIH and these maternal and child health factors, highlighting the importance of comprehensive prenatal and postnatal care.

**Conclusion:**

The study underscores the multifactorial nature of MIH, revealing significant associations with maternal illnesses, delivery complications, and early childhood health issues. These findings emphasize the need for targeted preventive measures and early interventions to manage MIH effectively, particularly in children at higher risk due to compromised maternal or child health.

## RESUMEN

**Objetivos:**

Este estudio tiene como objetivo investigar los factores que contribuyen a la hipomineralización de molares e incisivos (HMI), con un enfoque en la salud materna durante el embarazo, las complicaciones del parto y los factores de salud y nutrición posnatales en los niños.

**Métodos:**

Se realizó un estudio transversal que incluyó encuestas detalladas completadas por las madres sobre su salud durante el embarazo, las experiencias del parto y la historia de salud y nutrición temprana de sus hijos. Se realizaron exámenes clínicos a los niños para diagnosticar HMI según criterios establecidos. Los análisis estadísticos se realizaron utilizando IBM SPSS 29.0, incluidas estadísticas descriptivas y análisis de correlación para identificar asociaciones entre la HMI y varios factores de salud materna e infantil.

**Resultados:**

El estudio encontró que el 36,7% de las madres experimentaron enfermedades durante el embarazo, siendo la diabetes gestacional y la hipertensión las condiciones más comunes. Se observaron complicaciones durante el parto en el 3,3% de los casos, y el 16,7% de las madres tuvieron partos prematuros. Los problemas de salud comunes en los niños incluyeron infecciones respiratorias (26,7%), fiebre (23,3%) y otitis media (16,7%). Notablemente, los defectos dentales fueron más prevalentes en niños con trastornos renales (66,7%), trastornos del sistema digestivo (45,8%) y diarrea severa (43,8%). Se identificaron correlaciones significativas entre la HMI y estos factores de salud materna e infantil, lo que resalta la importancia de una atención prenatal y posnatal integral.

**Conclusión:**

El estudio subraya la naturaleza multifactorial de la HMI, lo que revela asociaciones significativas con enfermedades maternas, complicaciones del parto y problemas de salud en la infancia temprana. Estos hallazgos enfatizan la necesidad de medidas preventivas dirigidas e intervenciones tempranas para manejar la HMI de manera efectiva, particularmente en niños con mayor riesgo debido a la salud materna o infantil comprometida.

## INTRODUCTION

Molar Incisor Hypomineralization (MIH) is a developmental dental condition characterized by the presence of hypomineralized enamel in one to four first permanent molars, frequently associated with similarly affected permanent incisors.^1^^,^^2^ The condition manifests as well-defined opacities that vary in color from white to yellow-brown and often leads to post-eruptive enamel breakdown. This compromised enamel quality can result in increased tooth sensitivity, higher susceptibility to dental caries, and significant aesthetic concerns, which can affect a child’s overall well-being and quality of life.^2^^,^^3^

The etiology of MIH is multifactorial and not fully understood, with both genetic and environmental factors believed to play roles. Various prenatal, perinatal, and postnatal factors have been proposed as potential contributors to the development of MIH.^4^^,^^5^ These include maternal illnesses and stress during pregnancy, complications during delivery, and childhood illnesses and nutritional deficiencies. Understanding these contributing factors is crucial for developing preventive strategies and improving the management of MIH.^6^

Maternal health during pregnancy has been identified as a significant factor in the development of MIH. Studies suggest that maternal illnesses such as gestational diabetes, hypertension, and infections during pregnancy can adversely affect the developing enamel of the fetus.^7^^-^^9^ Additionally, complications during delivery, such as premature birth, may also contribute to the risk of MIH, as premature infants often experience a range of health challenges that can impact their dental development.^10^

Postnatal factors, including the child’s health and nutrition, are also critical in the development of MIH. Early childhood illnesses such as respiratory infections, fever, and gastrointestinal disturbances can disrupt normal enamel formation.^11^ Furthermore, nutritional deficiencies, particularly in essential vitamins and minerals like vitamin D and calcium, have been associated with a higher prevalence of MIH.8 Breastfeeding duration and the use of supplements in early childhood can influence the overall dental health of children.^12^

Despite the increasing recognition of MIH and its impact on dental health, there remains a need for more comprehensive studies to identify and confirm the various factors contributing to its development. By understanding these factors, dental professionals can better predict and manage MIH, ultimately improving the quality of care for affected children. The aim of this study was to investigate the demographic and clinical factors associated with the prevalence and severity of MIH in children. By examining maternal health during pregnancy, delivery complications, and postnatal health and nutritional factors, this study sought to identify key risk factors and provide insights into potential preventive measures for MIH.

## MATERIALS AND METHODS

Ethical approval for the study was obtained from the ethics committee (Gülhane Ethics Committee 14/1648.4-361), ensuring that the research adhered to the highest ethical standard. The study was conducted on patients who visited the Gülhane Pediatric Dentistry Department, Ankara Turkey. A total of 30 patients, including 12 females and 18 males, with molar-incisor hmineralization detected during routine examinations, were included in the study. Informed consent was obtained from all participants before data collection began, ensuring that participants were fully aware of the study's aims and their rights. The confidentiality and anonymity of all participants were strictly maintained throughout the study.

Surveys were administered to families to gather detailed information about maternal health during pregnancy, birth complications, and the child's postnatal health and nutritional history. These surveys were conducted through face-to-face interviews and were designed to ensure comprehensive data collection. Questions covered a range of topics, including any illnesses or infections the mother experienced during pregnancy, complications during this period, and the child's early health and nutrition, such as breastfeeding duration and the use of vitamin and mineral supplements (Table 1). Permanent molars and permanent incisors were examined and the findings recorded according with the recommended criteria for MIH diagnosis and recording (Weerheijm et al.^13^) by CY (Table 2). 

**Table 1:** MIH Survey

Question	Options			
1. Did you have any illness or infection during your pregnancy?	Yes No			
If yes, during which trimester did you have this illness?	First trimester Second trimester Last trimester			
If yes, what type of illness was it?	Hypocalcemia Vitamin D deficiency Hypertension Gestational diabetes Preeclampsia Other			
2. Did any complications occur during your child's birth?	Yes No			
If yes, what type of complication occurred?				
3. What was the mode of delivery for your child?	Vaginal delivery Cesarean section			
4. Was your child born prematurely?	Yes No			
If yes, at how many weeks was your child born?				
5. What was your child’s birth weight?	Less than 1.5 kg Between 1.5-2.5 kg More than 2.5 kg			
6. How long did you breastfeed your child?	Did not breastfeed Less than 12 months 12-18 months More than 12 months			
7. Did you give your child fluoride, calcium, or vitamin supplements in tablet, syrup, or drop form until the age of 4?	Yes No			
If yes, from what age, for how many months, and how many times per day did you give the supplements?				
8. Which of the following illnesses did your child experience until the age of 4? If they did, at what age and how frequently?				
Illness	Yes	No	Age	Frequency
Severe Diarrhea				
Otitis Media				
Asthma				
Pneumonia				
Lower Respiratory Tract Infection				
(Bronchitis, Bronchiolitis, Laryngitis)				
Tonsillitis/Pharyngitis				
Convulsions				
Fever				
Chronic Kidney Disease				
Digestive System Disorder				
Urinary Tract Infection				
Any Kidney Disorder				
Allergy				
				
Chickenpox				
Scarlet Fever				
Mumps				
Measles				

**Table 2:** MIH Index (Weerheijm et al.^19^, 2003)

Grade	Description
0	No defect
1	Demarcated opacities with white to cream color change
2	Demarcated opacities with yellow to brown color change
3a	Mild, initial substance loss
3b	Moderate, substance loss affecting dentin due to wear or caries
3c	Severe, extensive crown destruction due to wear or caries
4a	Restored with amalgam
4k	Restored with composite
4c	Restored with glass ionomer cement
4p	Restored with stainless steel crown
5	Tooth loss due to molar incisor hypomineralization
6	Exclude molars and incisors with less than half of the crown erupted

Data were collected from a cohort of mothers and their children. The study utilized the IBM Statistical Package for the Social Sciences (SPSS) for Macos 29.0 for analysis. Descriptive statistics, including frequency, percentage, mean, standard deviation, median, minimum, and maximum values, were calculated for both categorical and continuous variables.

## RESULTS

### Demographic and Clinical Characteristics of Mothers

As shown in Table 3, 36.7% (11 individuals) of the mothers included in the study experienced illness or infection during pregnancy. Of these, 30% (3 individuals) experienced the illness in the first trimester, another 30% (3 individuals) in the last trimester, and 40% (4 individuals) in both periods. The illnesses experienced included gestational diabetes (20%, 2 individuals) and hypertension (20%, 2 individuals), among others. Complications during delivery were noted in 3.3% (1 individual) of the cases, and 16.7% (5 individuals) of the mothers had premature deliveries. The average gestational age was 35 weeks, with a median of 37 weeks.

**Table 3:** Distribution of Mothers' Demographic and Clinical Characteristics

Variables	n (%)	Mean±SD	Median (Min-Max)
Illness or infection during pregnancy	11 (36.7)		
If yes, during which trimester did you have this illness?			
First trimester	3 (30)		
Last trimester	3 (30)		
Both	4 (40)		
Illnesses experienced			
Pulmonary edema	1 (10)		
Vitamin B deficiency	1 (10)		
Gestational diabetes	2 (20)		
Hypertension (HT)	2 (20)		
Heart disease	1 (10)		
Cystitis	1 (10)		
Thyroid	3 (30)		
Complication during delivery	1 (3.3)		
Mode of delivery			
Vaginal delivery	17 (56.7)		
Cesarean section	13 (43.3)		
Preterm birth	5 (16.7)		
Gestational age (weeks)		35±4	37 (28-38)

### Demographic and Clinical Characteristics of Children

Table 4 presents the demographic and clinical characteristics of the children. The ages of the children ranged from 6 to 15 years, with an average and median age of 10 years. Of the children, 40% (12 individuals) were female, and 60% (18 individuals) were male. A significant majority (83.3%, 25 individuals) had a birth weight greater than 2.5 kg. Breastfeeding duration varied, with 6.7% (2 individuals) not being breastfed, 46.7% (14 individuals) breastfed for up to 12 months, and another 46.7% (14 individuals) breastfed for more than 12 months. By the age of four, 38.9% (7 individuals) received vitamin D supplements, 5.6% (1 individual) received calcium, 38.9% (7 individuals) received iron drops, and 22.2% (4 individuals) received fluoride tablets. The most common illnesses experienced by the children were lower respiratory tract infections (26.7%, 8 individuals), fever (23.3%, 7 individuals), and otitis media (16.7%, 5 individuals).

**Table 4:** Distribution of Children's Demographic and Clinical Characteristics

Variables	n (%)	Mean±SD	Median (Min-Max)
Age (years)		10±3	10 (6-15)
Gender			
Female	12 (40)		
Male	18 (60)		
Birth weight			
Less than 1.5 kg	1 (3.3)		
1.5-2.5 kg	4 (13.3)		
More than 2.5 kg	25 (83.3)		
Duration of breastfeeding			
Not breastfed	2 (6.7)		
0-12 months	14 (46.7)		
More than 12 months	14 (46.7)		
Received fluoride, calcium, or vitamin supplements until age 4	18 (60)		
Vitamin D	7 (38.9)		
Calcium	1 (5.6)		
Iron drops	7 (38.9)		
Fluoride tablets	4 (22.2)		
Illnesses experienced			
Severe diarrhea	4 (13.3)		
Otitis media	5 (16.7)		
Asthma	1 (3.3)		
Pneumonia	1 (3.3)		
Lower respiratory tract infection	8 (26.7)		
Tonsillitis/Pharyngitis	3 (10)		
Fever	7 (23.3)		
Digestive system disorder	2 (6.7)		
Urinary tract infection	4 (13.3)		
Any kidney disorder	2 (6.7)		
Allergy	4 (13.3)		
Other illnesses			
Chickenpox	18 (60)		
Age at chickenpox		4±3	4 (1-12)
Scarlet fever	1 (3.3)		
Age at scarlet fever		3±0	3 (3-3)
Mumps	3 (10)		
Age at mumps		3±0	3 (3-3)

### Dental Disorders in Children

Table 5 and Table 6 detail the distribution of dental disorders according to the tooth number and illnesses experienced by the children. The most common dental disorder observed was clearly defined white to cream-colored opacities. Dental defects were most prevalent in children who had experienced kidney disorders, with 66.7% (16 teeth) showing defects, followed by those with digestive system disorders (45.8%, 11 teeth) and severe diarrhea (43.8%, 21 teeth). The lowest prevalence of defects was observed in children with asthma (16.7%, 2 teeth). 

**Table 5:** Distribution of Dental Defects by Tooth Number in Children

Dental Defect by Tooth Number	11 n (%)	16 n (%)	21 n (%)	26 n (%)	31 n (%)	36 n (%)	41 n (%)	46 n (%)	12 n (%)	22 n (%)	32 n (%)	42 n (%)
No defect	10 (33.3)	8 (26.7)	12 (41.4)	9 (30)	24 (80)	8 (26.7)	26 (86.7)	11 (36.7)	0 (0)	0 (0)	0 (0)	0 (0)
Opacities with well-defined white to cream color change	15 (50)	7 (23.3)	15 (51.7)	7 (23.3)	5 (16.7)	6 (20)	4 (13.3)	5 (16.7)	2 (66.7)	3 (75)	3 (100)	3 (100)
Opacities with well-defined yellow to brown color change	3 (10)	4 (13.3)	0 (0)	4 (13.3)	1 (3.3)	2 (6.7)	0 (0)	1 (3.3)	1 (33.3)	1 (25)	0 (0)	0 (0)
Mild, initial substance loss	0 (0)	1 (3.3)	0 (0)	1 (3.3)	0 (0)	2 (6.7)	0 (0)	2 (6.7)	0 (0)	0 (0)	0 (0)	0 (0)
Moderate, substance loss affecting dentin due to wear or caries	0 (0)	6 (20)	0 (0)	3 (10)	0 (0)	4 (13.3)	0 (0)	5 (16.7)	0 (0)	0 (0)	0 (0)	0 (0)
Severe, extensive crown destruction due to wear or caries	0 (0)	2 (6.7)	0 (0)	6 (20)	0 (0)	6 (20)	0 (0)	3 (10)	0 (0)	0 (0)	0 (0)	0 (0)
Restored with amalgam	1 (3.3)	2 (6.7)	1 (3.4)	0 (0)	0 (0)	2 (6.7)	0 (0)	3 (10)	0 (0)	0 (0)	0 (0)	0 (0)
Restored with composite	1 (3.3)	0 (0)	1 (3.4)	0 (0)	0 (0)	0 (0)	0 (0)	0 (0)	0 (0)	0 (0)	0 (0)	0 (0)
Restored with glass ionomer cement	0 (0)	0 (0)	0 (0)	0 (0)	0 (0)	0 (0)	0 (0)	0 (0)	0 (0)	0 (0)	0 (0)	0 (0)
Restored with stainless steel crown	0 (0)	0 (0)	0 (0)	0 (0)	0 (0)	0 (0)	0 (0)	0 (0)	0 (0)	0 (0)	0 (0)	0 (0)
Tooth loss due to molar incisor hypomineralization	0 (0)	0 (0)	0 (0)	0 (0)	0 (0)	0 (0)	0 (0)	0 (0)	0 (0)	0 (0)	0 (0)	0 (0)
Exclude molars and incisors with less than half of the crown erupted	0 (0)	0 (0)	0 (0)	0 (0)	0 (0)	0 (0)	0 (0)	0 (0)	0 (0)	0 (0)	0 (0)	0 (0)

**Table 6:** Distribution of Dental Defects by Illnesses in Children

Illnesses	Number of Patients	Opacities with Well-Defined White to Cream Color Change n (%)	Opacities with Well-Defined Yellow to Brown Color Change n (%)	Mild, Initial Substance Loss n (%)	Moderate, Substance Loss Affecting Dentin Due to Wear or Caries n (%)	Severe, Extensive Crown Destruction Due to Wear or Caries n (%)	Restored with Amalgam n (%)	Total n (%)
Severe Diarrhea	4	11 (22.9)	4 (8.3)	1 (2.1)	3 (6.3)	0 (0)	2 (4.2)	21 (43.8)
Otitis Media	5	8 (13.3)	4 (6.7)	2 (3.3)	6 (10)	1 (1.7)	3 (5)	24 (40)
Asthma	1	2 (16.7)	0 (0)	0 (0)	0 (0)	0 (0)	0 (0)	2 (16.7)
Pneumonia	1	2 (16.7)	0 (0)	0 (0)	1 (8.3)	0 (0)	1 (8.3)	4 (33.3)
Lower Respiratory Tract Infection	8	21 (21.9)	7 (7.3)	4 (4.2)	3 (3.1)	4 (4.2)	1 (1)	40 (41.7)
Tonsillitis/Pharyngitis	3	10 (27.8)	2 (5.6)	2 (5.6)	0 (0)	0 (0)	0 (0)	14 (38.9)
Fever	7	6 (7.1)	7 (8.3)	2 (2.4)	3 (3.6)	5 (6)	0 (0)	23 (27.4)
Digestive System Disorder	2	3 (12.5)	1 (4.2)	1 (4.2)	3 (12.5)	0 (0)	3 (12.5)	11 (45.8)
Urinary Tract Infection	4	7 (14.6)	1 (2.1)	0 (0)	2 (4.2)	0 (0)	4 (8.3)	14 (29.2)
Any Kidney Disorder	2	11 (45.8)	1 (4.2)	0 (0)	1 (4.2)	0 (0)	3 (12.5)	16 (66.7)
Allergy	4	9 (18.8)	1 (2.1)	2 (4.2)	2 (4.2)	0 (0)	4 (8.3)	18 (37.5)
Chickenpox	18	47 (21.8)	8 (3.7)	3 (1.4)	15 (6.9)	4 (1.9)	7 (3.2)	84 (38.9)
Scarlet Fever	1	0 (0)	2 (16.7)	2 (16.7)	0 (0)	0 (0)	0 (0)	4 (33.3)
Mumps	3	2 (5.6)	4 (11.1)	3 (8.3)	0 (0)	1 (2.8)	0 (0)	10 (27.8)

## DISCUSSION

The findings of this study provide significant insights into the correlations between prenatal and postnatal health factors and the development of Molar Incisor Hypomineralization (MIH). Our results are consistent with several other studies in the literature, which have also identified a range of maternal and early childhood health issues as potential contributors to MIH.^14^^-^^18^

A notable aspect of our study is the high prevalence of maternal illnesses during pregnancy. We found that 36.7% of the mothers experienced illnesses or infections, with gestational diabetes and hypertension being particularly common. This aligns with the findings of Elfrink et al.^14^, who reported that maternal illnesses such as fever and infections during pregnancy are associated with an increased risk of MIH in children. Similarly, a study by Alaluusua et al.^15^ highlighted the potential role of maternal health and stress during pregnancy in the etiology of MIH.

Our study also identified obstetric complications and preterm births as significant factors, with 3.3% of the cases involving obstetric complications and 16.7% of the mothers having preterm deliveries. This is in agreement with the research of Lygidakis et al.^16^, which found that preterm birth and related complications could adversely affect enamel development, increasing the risk of MIH. The average gestational age in our study was 35 weeks, with a median of 37 weeks, suggesting that premature birth is a critical period for enamel formation and subsequent MIH development.

In terms of early childhood health, our findings that lower respiratory tract infections, fever, and otitis media are common among the affected children are consistent with previous research. Jälevik and Norén^17^ reported that systemic conditions, particularly respiratory diseases and high fever during early childhood, are linked to MIH. These health issues can disrupt enamel mineralization processes during critical periods of dental development.

Nutritional deficiencies also emerged as a significant factor in our study. By the age of four, a considerable number of children in our cohort had received vitamin D, calcium, and iron supplements, reflecting concerns about nutritional status. This supports the conclusions of Fatturi et al.^17^, who emphasized the role of adequate nutrition, especially the intake of essential minerals and vitamins, in preventing hypomineralization. The deficiencies in vitamin D and calcium identified in our study are particularly relevant, given their known impact on enamel formation and mineralization.

Our analysis of dental defects revealed that children with a history of kidney disorders, digestive system disorders, and severe diarrhea had the highest prevalence of Molar Incisor Hypomineralization (MIH). Kidney disorders, in particular, have a profound impact on calcium and phosphate metabolism, which are critical for proper enamel mineralization. Disruptions in these metabolic processes can lead to hypocalcemia, directly affecting the development of dental enamel during critical periods of tooth formation. This finding is consistent with previous studies, such as those by Ghanim et al.^18^, which reported a significant association between systemic health conditions and the occurrence of MIH. Children suffering from kidney disorders may experience chronic imbalances in essential minerals, leading to weakened enamel structure and increased susceptibility to MIH. Additionally, digestive system disorders, particularly those involving malabsorption, can limit the availability of essential nutrients such as calcium, vitamin D, and phosphate, further exacerbating the risk of MIH. Severe diarrhea, often a symptom of underlying gastrointestinal issues, can lead to acute and chronic nutritional deficiencies, compounding the enamel's vulnerability to hypomineralization. These systemic health conditions create a multifactorial challenge in dental health, emphasizing the need for targeted medical and nutritional interventions to mitigate the risk of MIH in vulnerable pediatric populations. Our findings reinforce the importance of early diagnosis and comprehensive management of these systemic conditions to prevent or minimize the impact of MIH on children's dental health.

Comparing our results with the broader literature highlights the multifactorial nature of MIH. While our study reinforces the established understanding of MIH etiology, it also underscores the need for a comprehensive approach to prenatal and postnatal care to mitigate these risk factors. Effective management of maternal health conditions during pregnancy, prevention of preterm births, and ensuring adequate nutrition during early childhood are essential strategies for reducing the incidence of MIH.

## CONCLUSION

This study highlights significant associations between maternal health during pregnancy, delivery complications, and early childhood health factors with the prevalence and severity of Molar Incisor Hypomineralization (MIH). These findings underscore the multifactorial nature of MIH and the importance of comprehensive prenatal and postnatal care. Targeted preventive measures and early interventions are crucial in reducing the risk of MIH and improving dental health outcomes in children.

## Financing:

This research did not receive any specific grant from funding agencies in the public, commercial, or not-for-profit sectors.
